# A Crystal Plasticity Phase-Field Study on the Effects of Grain Boundary Degradation on the Fatigue Behavior of a Nickel-Based Superalloy

**DOI:** 10.3390/ma18143309

**Published:** 2025-07-14

**Authors:** Pengfei Liu, Zhanghua Chen, Xiao Zhao, Jianxin Dong, He Jiang

**Affiliations:** 1 School of Mathematics and Physics, University of Science and Technology Beijing, 30 Xueyuan Road, Haidian District, Beijing 100083, China; liupengfei94@126.com (P.L.); chenzhanghua@ustb.edu.cn (Z.C.); 2 Beijing Advanced Innovation Center for Materials Genome Engineering, University of Science and Technology Beijing, 30 Xueyuan Road, Haidian District, Beijing 100083, China; 3 School of Materials Science and Engineering, University of Science and Technology Beijing, 30 Xueyuan Road, Haidian District, Beijing 100083, China; zhaoxiao78@163.com (X.Z.); jxdong@ustb.edu.cn (J.D.)

**Keywords:** crystal plasticity, phase-field model, fatigue crack propagation, grain boundary oxidation

## Abstract

Grain boundary weakening in high-temperature environments significantly influences the fatigue crack growth mechanisms of nickel-based superalloys, introducing challenges in accurately predicting fatigue life. In this study, a dislocation-density-based crystal plasticity phase-field (CP–PF) model is developed to simulate the fatigue crack growth behavior of the GH4169 alloy under both room and elevated temperatures. Grain boundaries are explicitly modeled, enabling the competition between transgranular and intergranular cracking to be accurately captured. The grain boundary separation energy and surface energy, calculated via molecular dynamics simulations, are employed as failure criteria for grain boundary and intragranular material points, respectively. The simulation results reveal that under oxygen-free conditions, fatigue crack propagation at both room and high temperatures is governed by sustained shear slip, with crack advancement hindered by grains exhibiting low Schmid factors. When grain boundary oxidation is introduced, increasing oxidation levels progressively degrade grain boundary strength and reduce overall fatigue resistance. Specifically, at room temperature, oxidation shortens the duration of crack arrest near grain boundaries. At elevated service temperatures, intensified grain boundary degradation facilitates a transition in crack growth mode from transgranular to intergranular, thereby accelerating crack propagation and exacerbating fatigue damage.

## 1. Introduction

Nickel-based superalloys are widely employed in critical rotating components of aeroengines, such as turbine disks, owing to their excellent high-temperature fatigue strength and creep resistance. Under complex thermo-mechanical loading conditions, fatigue failure is the dominant failure mode for these alloys during long-term service. This process typically involves microcrack initiation, stable crack propagation, and final instantaneous fracture [1,2,3,4]. Therefore, developing numerical models capable of capturing multi-scale damage evolution mechanisms is of great significance for improving the accuracy of fatigue life prediction and enhancing the service reliability of these materials.

The fatigue performance of nickel-based alloys is governed by a range of complex factors, including load amplitude, cyclic frequency, microstructural features such as inclusions or voids, and the service environment [5,6,7,8,9,10]. Among these, temperature is a critical parameter that influences both the fatigue failure mechanism and the crack propagation rate. Numerous studies have demonstrated that the fatigue life of superalloys gradually decreases with increasing temperature, with a sharp decline occurring once the temperature exceeds typical service conditions [11,12,13]. Further experimental investigations have validated the effect of temperature on the transition of fatigue crack propagation modes. For instance, Xu et al. proposed a “transgranular-to-intergranular transition threshold” ($∆K_{T}$), which defines the stress intensity factor at which cracks shift from transgranular to intergranular propagation. In the FGH4097 alloy, $∆K_{T}$ decreases significantly as temperature increases: fatigue cracks propagate transgranularly at room temperature and 400 °C, while a transition to intergranular propagation occurs between 650 °C and 800 °C. Correspondingly, $∆K_{T}$ values drop from 59.0 $MPa\cdot m^{1/2}$ to 28.5 $MPa\cdot m^{1/2}$ [14,15]. Similar trends have also been reported in alloys such as GH4738, FGH4096, FGH4098, and Inconel 718 [16,17].

The shift in fatigue crack propagation mode induced by elevated temperatures fundamentally originates from differences in damage evolution mechanisms at varying temperature regimes. Under room-temperature cyclic loading, damage typically accumulates through persistent slip bands (PSBs), which serve as preferential sites for microcrack initiation and early propagation. Using in-situ techniques such as transmission electron microscopy (TEM) and electron backscatter diffraction, Guo et al. provided direct evidence that dislocation activity is highly localized within PSBs and that these structures act as critical precursors to fatigue crack formation [18]. Similarly, Yang et al. demonstrated in high-cycle fatigue experiments on Inconel 718 that non-metallic inclusions promote localized dislocation pile-up and PSB formation, ultimately leading to microcrack nucleation [19]. As understanding of crack initiation mechanisms has deepened, numerical modeling techniques have evolved rapidly. The crystal plasticity finite element method (CPFEM) has been widely adopted to compute microscopic mechanical field variables—such as accumulated plastic strain, shear strain, and local stored energy density—that are closely associated with the evolution of PSBs. These variables serve as fatigue indicator parameter, enabling high-accuracy prediction of microcrack initiation and fatigue life [20,21,22,23,24,25,26]. To overcome the limitations of traditional CPFEM in directly capturing material degradation caused by damage accumulation, researchers have coupled CPFEM with fracture mechanics–based approaches, such as cohesive zone models (CZM), extended finite element methods (XFEM), and phase-field fracture methods. For example, Han et al. successfully simulated surface microcrack initiation and early propagation in AISI 52100 bearing steel under rolling contact fatigue by integrating CPFEM with CZM [27]. Xu et al. developed a mesoscopic structure–function model coupling CPFEM with damage to investigate the correlation between accumulated plastic strain and damage evolution. Long et al. coupled CPFEM with XFEM, employing local stored energy density as the driving force for crack propagation to study microstructure-sensitive fatigue crack growth in Zircaloy-4 [28,29]. Cheng et al. proposed a model combining CPFEM with void damage theory to capture the complex interactions between polycrystalline microstructures and porosity behavior [30]. Considering the advantages of the phase-field fracture method in simulating crack driving forces and topological evolution, we previously developed a CPFEM–PFM coupled model that demonstrated excellent capability in simulating fatigue crack propagation in superalloys at room temperature, with high sensitivity to crystallographic features such as grain orientation, grain size, and misorientation [31].

Elevated temperatures significantly complicate the mechanisms of fatigue crack propagation. In addition to the fatigue damage caused by dislocation slip, temperature-induced degradation of grain boundary cohesion and grain boundary oxidation-induced embrittlement have become major contributors to fatigue damage accumulation. These mechanisms collectively accelerate the nucleation and propagation of intergranular cracks and significantly reduce high-temperature fatigue life [32,33,34,35]. Consequently, numerical models must account for the competing effects of dislocation slip and interfacial degradation. The CZM–CPFEM coupled framework has been widely employed in grain boundary decohesion studies due to its robust capability in characterizing interfacial debonding behavior. For instance, Gonzalez et al. introduced cohesive elements at grain boundary locations within a polycrystalline model to investigate the influence of intergranular stress on grain boundary damage and stress corrosion cracking mechanisms [36]. Wang et al. defined the energy required to fail three-dimensional cohesive elements at grain boundaries within a CZM–CPFEM framework, successfully simulating grain boundary decohesion. Their approach accurately predicted fracture characteristics of TWIP steels from stress–strain curves and strain hardening rates [37]. Besides, other damage mechanics or fracture mechanics–based methods have also been combined with CPFEM to study complex fatigue damage and cracking mechanisms. Salvini et al. employed a CPFEM–phase field fracture model to investigate the evolution of intragranular and intergranular damage in 316H austenitic stainless steel during the early stage of fatigue [38]. Zhao et al. implemented a custom damage parameter to degrade the stiffness of integration points, thereby modeling the failure process of material points. They established the first CPFEM-based model incorporating creep–fatigue and oxidation-assisted fracture mechanisms to capture the transgranular-to-intergranular crack mode transition at elevated temperatures [39]. Considering that the phase field fracture method does not require predefined crack paths or special elements and naturally accommodates complex crack topologies [40,41,42], the present study adopts a CPFEM–PFM coupled model as the modeling framework. Based on our previous work, this model is further extended by incorporating modifications for high-temperature compatibility in the crystal plasticity constitutive relations. Explicit grain boundary structures are introduced, and distinct energy-based failure criteria are assigned to interior grain points and grain boundary regions. Specifically, (111) plane surface energy and grain boundary separation energy—obtained via molecular dynamics simulations—are respectively used to determine the failure of grain interiors and grain boundaries, thereby capturing the degradation of interfacial toughness at elevated temperatures and under various oxidation levels. This framework enables the competition between transgranular and intergranular fracture modes to be realistically simulated.

In summary, this study proposes a crystal plasticity–phase field coupled model based on dislocation density theory to simulate the fatigue crack propagation behavior of nickel-based superalloys under varying temperatures and grain boundary oxidation levels. Section 2 introduces the experimental procedures and reviews relevant studies on high-temperature fatigue behavior. Section 3 presents the governing equations of the model, the grain boundary modeling strategy, and the molecular dynamics simulations used to determine energy-based failure criteria. Section 4 discusses the numerical results of crack propagation and analyzes the mechanisms by which temperature and grain boundary oxidation affect fatigue crack growth. Section 5 concludes this study by summarizing the key findings.

## 2. Material and Experiments

### 2.1. Materials and Aging Treatments

The material investigated in this study is GH4169, a precipitation-hardened nickel-based superalloy. Owing to its exceptional high-temperature strength, oxidation resistance, and microstructural stability, GH4169 is extensively used in critical hot-section components of aeroengines, such as turbine disks [43,44,45]. Its nominal chemical composition (in weight percent) is as follows: Ni 52.77, Cr 19.30, Fe (balance), Nb 5.34, Mo 3.01, Ti 1.10, Al 0.40, C 0.026, Mn 0.020, P 0.009, and B 0.004. The alloy is manufactured through a combination of vacuum induction melting, electroslag remelting, and vacuum arc remelting, followed by standard solution treatment and aging. This processing route produces a characteristic dual-phase γ′/γ″ precipitation-hardened microstructure, which enables the alloy to retain excellent mechanical properties under elevated-temperature service conditions.

With increasing temperature and prolonged service duration, oxidation-induced damage in the material progressively intensifies. To simulate the long-term degradation behavior experienced under high-temperature operating conditions, specimens were thermally exposed to air at 650 °C—a representative service temperature—for durations of 1000, 3000, and 5000 h. This thermal exposure was designed to replicate the microstructural damage accumulation caused by oxidation during extended service and to generate samples exhibiting varying degrees of grain boundary oxidation.

### 2.2. Fatigue Crack Growth Experiments

**Figure 1:**
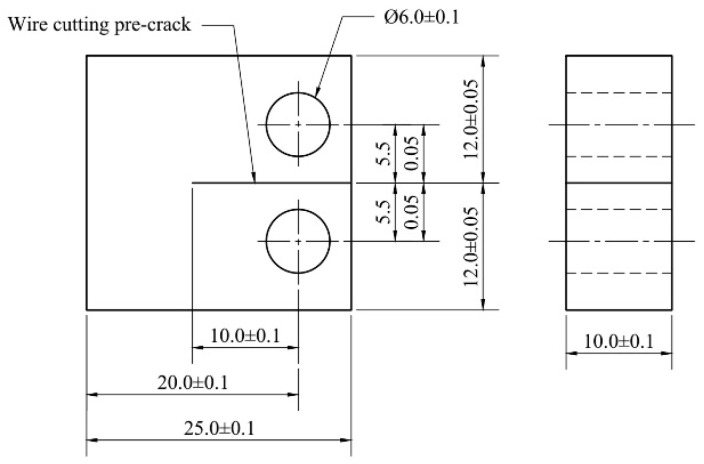
Schematic diagram of the compact tension (CT) specimen geometry [31]. Reprinted with permission from Ref. [31]. Copyright 2024 Elsevier.

Fatigue crack growth experiments were conducted on GH4169 alloy specimens in both the unaged condition and after thermal aging at various temperatures and durations. The tests were performed using a CMT5204GLW high-precision fatigue testing machine (Sansi Taijie, Zhuhai, China). Standard compact tension (CT) specimens were employed, with dimensions of 25 × 24 × 10 mm and an initial pre-crack of 10 mm introduced via electrical discharge machining (EDM), as illustrated in Figure 1. The stress intensity factor range ∆K was calculated using the same formulation as in our previous work [14,31], in which compact tension (CT) specimens of identical geometry were used. This ensures the consistency and validity of the geometric coefficients in Equation (1).

(1) $$∆K=\frac{∆P}{B\cdot W^{1/2}}\left[29.6{\left(\frac{a}{W}\right)}^{1/2}-185.5{\left(\frac{a}{W}\right)}^{3/2}+655.7{\left(\frac{a}{W}\right)}^{5/2}-1017{\left(\frac{a}{W}\right)}^{7/2}+638.9{\left(\frac{a}{W}\right)}^{9/2}\right].$$

Here, a denotes the crack length, ∆P is the applied load range, and W and *B* represent the specimen width and thickness, respectively. All experiments were performed at room temperature (25 ± 5 °C) to eliminate the effects of real-time oxidation at the crack tip under elevated temperatures. This ensured that the observed crack growth behavior primarily reflected the damage accumulated during prior thermal exposure due to oxidation. A constant-amplitude loading mode was employed, with a stress ratio of R = 0.05, a maximum load of 5650 N, and a loading frequency of approximately 1/36 Hz. Crack length was continuously monitored using the direct current potential drop method, with the calibration relationship given by ∆a=7.89∆V.

**Figure 2:**
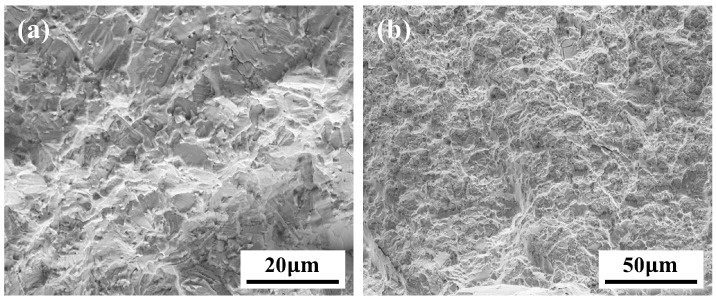
(**a**) Fatigue fracture of an unaged specimen. (**b**) Fatigue fracture of a specimen after aging at 650 °C for 1000 h.

Fractographic analysis was conducted using scanning electron microscopy (SEM) to identify the transition region where the fatigue crack propagation mode changes from transgranular to intergranular, referred to as the “intergranular transition point” ($∆K_{T}$). In the unaged condition, the transition point $∆K_{T}$ was measured to be 40.54 $MPa\cdot m^{1/2}$. After thermal exposure at 650 °C for 1000, 3000, and 5000 h, $∆K_{T}$ decreased to 39.82, 37.81, and 36.68 $MPa\cdot m^{1/2}$, respectively. Figure 2a,b show the fracture surfaces near the initial crack tip for the unaged specimen and the specimen thermally aged for 5000 h, respectively. The unaged specimen exhibits a relatively smooth fracture surface, characterized by transgranular features such as river patterns, cleavage steps, and clearly defined fatigue striations. In contrast, the specimen subjected to prolonged thermal aging displays a predominantly intergranular fracture morphology emerging from the early stages of crack propagation. The fracture surface becomes markedly rougher and reveals more pronounced grain boundary decohesion features, indicating a substantial shift in the crack propagation mechanism attributable to grain boundary degradation.

**Figure 3:**
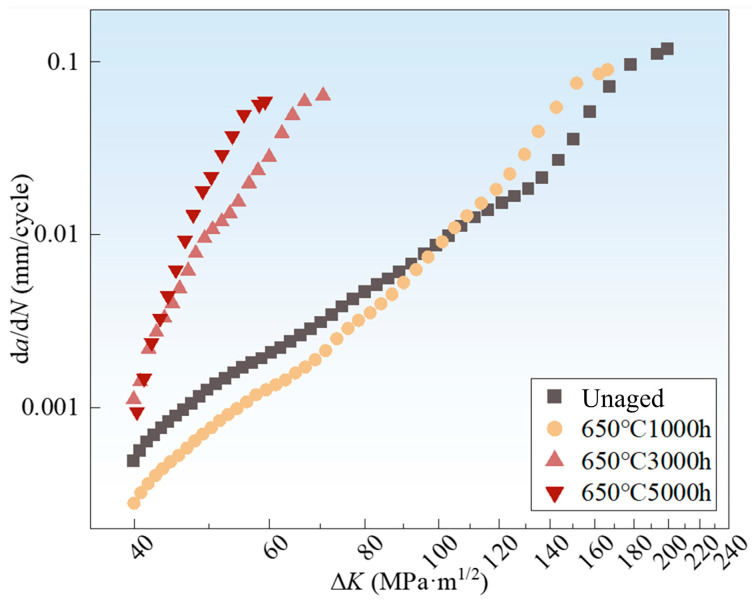
da/dN-ΔK curves for different aging durations at 650 °C.

As shown in Figure 3, the fatigue crack growth rate (da/dN) increases significantly with extended thermal aging duration. The specimen aged for 1000 h at 650 °C exhibited a relatively slow crack propagation rate during the early stages, likely due to a strengthening effect associated with precipitate coarsening. In contrast, under the 5000-h aging condition at 650 °C, the fatigue life of the specimen was reduced to approximately 25% of that of the unaged specimen, which indicates that prolonged oxidation at elevated temperatures significantly accelerates grain boundary degradation and markedly diminishes the alloy’s resistance to fatigue crack growth.

## 3. Modeling and Numerical Computation

This chapter outlines the development of the crystal plasticity–phase field (CP–PF) model. It begins by presenting the fundamental theoretical formulations that underpin the coupled CP–PF framework. Based on the microcrystallographic characteristics of the GH4169 alloy, a crystal-scale model is constructed with explicit representation of physical grain boundaries. Surface energies and grain boundary separation energies at various temperatures are obtained through molecular dynamics simulations. These values are employed as key damage-related parameters for grain interior and grain boundary regions, respectively. Finally, the model’s implementation within a finite element framework is described, including the procedures used to couple the crystal plasticity and phase field components.

### 3.1. Crystal Plasticity Phase-Field Model

#### 3.1.1. Crystal Plasticity Intrinsic Framework

The coupled crystal plasticity–phase field model (CP–PFM) is implemented in Abaqus/Standard through a two-layer computational framework. Abaqus is a widely adopted finite element software in CPFEM studies, offering high flexibility and extensibility through user-defined subroutines such as UMAT. This makes it an effective platform for implementing complex constitutive models, such as the elastic-plastic deformation and damage evolution formulations used in this study. In the first layer, the crystal plasticity module computes the elastoplastic deformation and microstructural responses, including the evolution of dislocation density. The kinematic decomposition of the deformation gradient F can be expressed in terms of elastic $F^{e}$ and plastic $F^{p}$ parts, as described in [46]:

(2) $$F=\frac{\partial x}{\partial X}F^{e}F^{p},$$

where the elastic deformation gradient $F^{e}$ describes the rigid body rotation and stretching of the lattice, and plastic deformation gradient $F^{p}$ contributes to the movements of dislocations on slip planes. The plastic rate gradient $L^{p}$ is contributed by the slip rate ${\dot{\gamma }}^{\alpha }$ of the activated α-th slip system, which can be written as follows [46]:

(3) $$L^{p}={\dot{F}}^{p}F^{p-1}=\sum_{\alpha =1}^{N_{s}}\left({\dot{\gamma }}^{\alpha }m^{\alpha }⊗n^{\alpha }\right),$$

where $N_{s}$ is the number of active slip systems, and the unit vectors $m^{\alpha }$ and $n^{\alpha }$ are parallel to the slip direction and normal direction of the slip plane of the slip system respectively. The symbol ⊗ denotes a dyadic product of tensors. The slip rate ${\dot{\gamma }}^{\alpha }$ on α-th slip system is expressed by a flow rule [47]:

(4) $${\dot{\gamma }}^{\alpha }={\dot{\gamma }}_{0}{\left[\frac{\left|\tau^{\alpha }\right|}{\tau_{CRSS}^{\alpha }}\right]}^{\frac{1}{m}}sign\left(\tau^{\alpha }\right),$$

where ${\dot{\gamma }}_{0}$ and *m* are material parameters representing the reference shearing rate and the rate sensitivity of slip, $\tau_{CRSS}^{\alpha }$ the critically resolved shear stress on the slip system α. The resolved shear stress, $\tau^{\alpha }$, on the α-th slip system is given as

(5) $$\tau^{\alpha }=F^{eT}F^{e}S:\left(m^{\alpha }⊗n^{\alpha }\right),$$

(6) $$S=C^{e}:\varepsilon^{e},$$

where S is the second PiolaKirchhoff stress, $\varepsilon^{e}$ is the elastic stiffness tensor, and $C^{e}$ represents the fourth-order elastic tensor. In the CPFE framework, the elastic response of the FCC-structured GH4169 alloy is characterized using three independent single-crystal elastic constants: $C_{11}$, $C_{12}$, and $C_{44}$.

The critically resolved shear stress $\tau_{CRSS}^{\alpha }$, is given in:

(7) $$\tau_{CRSS}^{\alpha }=\tau_{c0}+\mu b\sqrt{\;\rho_{total}},$$

where μ is the shear modulus, b is the magnitude of the Burgers vector, $\tau_{c0}$ represents the initial slip resistance. $\rho_{total}$ is the total dislocation density, which can be further divided into statistically stored dislocation (SSD) density and geometrically necessary dislocation (GND) density. The statistically stored dislocation (SSD) density is commonly used to characterize isotropic hardening behavior resulting from random dislocation interactions. A widely adopted approach is to assume a linear relationship between the SSD density and the accumulated equivalent plastic strain [48], expressed as:

(8) $${\dot{\rho }}_{SSD}=\lambda {\dot{\bar{\varepsilon }}}_{p}.$$

Here, λ is a proportionality constant, and ${\dot{\bar{\varepsilon }}}_{p}$ represents the growth rate of equivalent plastic strain.

Geometrically necessary dislocations (GNDs) are dislocations induced by geometric discontinuities or local crystallographic orientation changes—such as grain boundaries and slip bands—during plastic deformation. They reflect local strain gradients within the material and contribute to localized hardening. The scalar value of the total GND density can be expressed as [47]:

(9) $$\rho_{GND}=\sqrt{\sum_{\alpha =1}^{12}\left[{\left(\rho_{Gs}^{\alpha }\right)}^{2}+{\left(\rho_{Get}^{\alpha }\right)}^{2}+{\left(\rho_{Gen}^{\alpha }\right)}^{2}\right]},$$

where $\rho_{Gs}^{\alpha }$ is the pure screw component in the slip direction $m^{\alpha }$, $\rho_{Gen}^{\alpha }$ and $\rho_{Get}^{\alpha }$ are the pure edge components in the directions of the slip surface normal vectors $n^{\alpha }$ and $t^{\alpha }=n^{\alpha }\times m^{\alpha }$. They can be calculated by the following equation [49]:

(10) $${\dot{\rho }}_{Gs}^{\alpha }m^{\alpha }+{\dot{\rho }}_{Get}^{\alpha }t^{\alpha }+{\dot{\rho }}_{Gen}^{\alpha }n^{\alpha }=\frac{1}{b}curl\left({\dot{\gamma }}^{\alpha }n^{\alpha }F^{p}\right).$$

In previous studies, when the tensile part of the elastic energy density and the crystallographic energy were used as the driving forces for crack propagation, the simulated fatigue cracks were found to be most consistent with experimental observations [31]. Their corresponding formulations are as follows:

(11) $$W_{e}^{+}\left(\varepsilon \right)=\varepsilon_{+}^{e}:C:\varepsilon_{+}^{e},\;with\;\varepsilon_{+}^{e}=\sum_{i=1}^{3}{\langle \varepsilon_{i}^{e}\rangle }_{+}n_{i}⊗n_{i},$$

(12) $$W_{p}=\sum_{\alpha }\int_{0}^{t}\tau^{\alpha }{\dot{\gamma }}^{\alpha }dt.$$

Here, ${\langle \varepsilon_{i}^{e}\rangle }_{+}$ and $n_{i}$ represent the positive eigenvalues and the corresponding eigenvectors of the Eulerian logarithmic elastic strain tensor, respectively, while C denotes the fourth-order anisotropic elastic stiffness tensor accounting for cubic symmetry in the lattice. Considering the irreversibility of damage evolution, a history-dependent reference field H is defined to ensure the unidirectional nature of crack growth, expressed as [50]:

(13) $$H=\underset{T\in [0,t]}{\max}\left(W_{e}^{+}\right)+W_{p}.$$

The updated history reference field H is introduced into the phase-field calculation as the driving force for the phase-field evolution. The elastic and plastic parameters used in the crystal plasticity model, along with their calibration procedure based on experimental data, are detailed in Appendix A.

Table 1 shows the elastic and plastic material parameters used in the crystal plasticity finite element simulations conducted in this study.

#### 3.1.2. Phase Field Method

**Figure 4:**
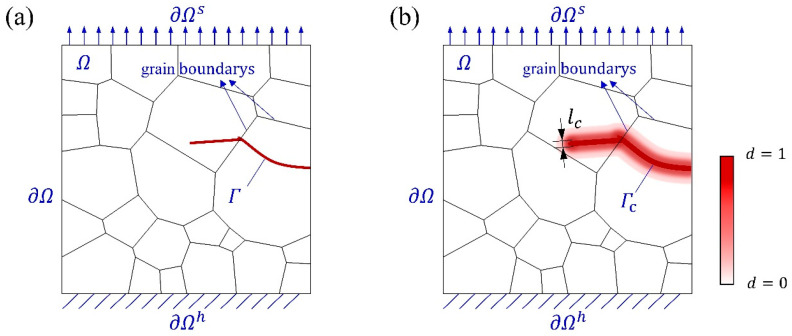
(**a**) Schematic diagram of a solid with an internal discontinuity boundary Γ. (**b**) Approximation of the phase field d to the internal discontinuity boundary.

Regarding a polycrystalline body Ω, whose external boundary is denoted as ∂Ω. This external boundary is further divided into the Dirichlet boundary $\partial \Omega^{h}$ and the Neumann boundary $\partial \Omega^{s}$, as described by $\partial \Omega =\partial \Omega^{s}+\partial \Omega^{h}$ and $\partial \Omega^{s}\cap \partial \Omega^{h}=0$. Additionally, the discrete crack Γ within Ω represents an internal boundary, as illustrated in Figure 4a. To avoid discontinuities associated with crack propagation tracking, the crack surface is instead represented by a phase field variable d, whose width corresponds to a finite internal length scale (Figure 4b). The phase field d∈[0,1] is a smooth, continuous scalar function used to describe the degree of material damage. In this formulation, d=1 corresponds to complete failure, and values between 0 and 1 represent the transition zone between the undamaged and fully fractured states. Crack evolution is captured by progressively degrading the material stiffness at each point, typically governed by a quadratic degradation function [51]:

(14) $$g\left(d\right)={\left(1-d\right)}^{2}+k.$$

The residual numerical stiffness k is typically assigned a small positive value close to zero to avoid singularity in the mechanical equilibrium equations. In this study, k is set to 0.001 to enhance convergence during finite element computations.

The crack surface density is calculated as [52]:

(15) $$\Gamma_{c}\left(d,\nabla d\right)=\frac{1}{2l_{c}}\left({l_{c}}^{2}\nabla d\cdot \nabla d+d^{2}\right),$$

where $l_{c}$ is the length scale parameter associated with the regulation of sharp discontinuities.

For ductile fracture mechanisms, it is essential to consider not only the balance and evolution between elastic strain energy and fracture energy but also the contribution of plastic deformation energy to the total potential energy of the system [53]. Accordingly, the total free energy density of an elastoplastic material can be expressed as [50]:

(16) $$W=W_{e}+W_{p}+W_{f},$$

where, $W_{e}$ and $W_{p}$ represent the elastic strain energy and the plastic stored strain energy per unit volume, corresponding to Equations (11) and (12), respectively. $W_{f}$ denotes the fracture energy density, which is given by the following expression:

(17) $$W_{f}=\int_{\Gamma }G_{c}d\Gamma =\int_{\Omega }\frac{G_{c}}{2l_{c}}\left({l_{c}}^{2}{\left|\nabla d\right|}^{2}+d^{2}\right)d\Omega ,$$

where, $G_{c}$ is the critical energy release rate, representing the energy required to generate a unit area of new fracture surface. The phase-field variable *d* typically evolves based on the balance between stored energy and fracture energy. By combining Equations (15)–(17), and replacing the phase-field driving force $\left(W_{e}+W_{p}\right)$ with a historical reference field H computed from the CPFE model, the total potential energy of the solid is obtained as:

(18) $$\Pi =\int_{\Omega }\left[g\left(d\right)H+\frac{G_{c}}{2l_{c}}\left({l_{c}}^{2}{\left|\nabla d\right|}^{2}+d^{2}\right)\right]d\Omega .$$

The phase-field model is implemented using a user-defined element (UEL) subroutine in the commercial finite element software ABAQUS (version 2022). Specifically, Equations (17) and (18) presented in this subsection are directly taken from our previous work [31], where the detailed formulations and derivations can be found. In this implementation, the UEL nodes are aligned with those of the CPE4 elements, which are four-node continuum plane strain elements widely used in finite element modeling, to ensure displacement field continuity within the CPFE framework. The phase-field variable, which characterizes crack evolution, is introduced as an additional nodal degree of freedom. Body forces are neglected, and the coupled displacement and phase-field equations are solved simultaneously.

### 3.2. Polycrystalline Models

In this study, the crystal model was constructed using the open-source software Neper (version 4.5.1), which employs the Voronoi method—a widely adopted approach in crystal plasticity finite element modeling. This technique generates polygonal elements to simulate the grain structure of polycrystalline materials and has proven effective in accurately reproducing the initial microstructural morphology and grain distribution of real alloys [54]. Based on typical grain sizes reported for GH4169 superalloys used in turbine disk applications—such as average grain sizes of 14.0 μm and 15.6 μm observed by Yuan et al. and Wang et al., respectively [55,56]—a crystal model with an average grain size of 15 μm was employed in this work to qualitatively simulate the fatigue crack growth behavior of GH4169 under uniaxial cyclic loading.

In previous studies employing the crystal plasticity finite element method (CPFEM), grain boundaries are often simplified as zero-thickness geometric interfaces. Integration points on either side of a shared boundary are assigned distinct Euler angles corresponding to their respective grains. This approach effectively captures deformation incompatibility between adjacent grains, resulting in localized dislocation accumulation and stress concentration, thereby providing a plausible explanation for damage localization near grain boundaries [57,58,59]. However, this simplification neglects the intrinsic differences in microstructural properties between grain boundaries and grain interiors. Such an omission becomes insufficient when grain boundary–specific mechanisms—such as decohesion or oxidation-induced weakening—play a dominant role. Given the present study’s focus on high-temperature and oxidation-induced grain boundary degradation and its effect on fatigue crack propagation, explicit modeling of grain boundaries and the incorporation of distinct failure mechanisms for intergranular and transgranular regions are essential.

Explicit grain boundary modeling has been extensively employed in simulations of intergranular fracture, particularly through the use of cohesive zone elements. For instance, the aforementioned CZM–CPFE-based studies have adopted this approach [36,37]. Notably, Grilli et al. developed a plugin that enables batch insertion of zero-thickness cohesive elements at grain boundary locations, significantly improving the efficiency of implementing cohesive zone models in intergranular fracture simulations [60]. An alternative strategy involves modeling grain boundaries as regions of finite thickness with material properties distinct from those of the grain interiors. For example, Zheng et al. introduced grain boundaries with constant thickness and assigned them isotropic properties, in contrast to the anisotropic behavior of intragranular elements governed by crystal plasticity. This configuration enabled the simulation of polycrystalline fracture under both tensile and compressive loading; under tensile conditions, the model successfully captured the coexistence of transgranular and intergranular cracking [61]. Similarly, Petkov et al. proposed a multiscale CPFE framework incorporating interface elements to represent grain boundary sliding, and demonstrated its effect on high-temperature creep behavior [62]. In a study on hydrogen-induced grain boundary embrittlement, Li et al. implemented a modeling scheme in which grain boundaries were explicitly represented by two layers of finite-thickness elements. These layers were assigned Euler angles identical to those of the adjacent grains, preserving the crystallographic specificity of each boundary and enabling accurate simulation of intergranular crack propagation [63].

Compared with zero-thickness modeling approaches, explicit grain boundary representation provides significantly greater flexibility for assigning customized material properties and failure mechanisms to grain boundaries. This approach not only enables a more accurate representation of the mechanical property differences between grain boundaries and grain interiors, but also establishes a more robust theoretical foundation for investigating the influence of temperature on fatigue failure mechanisms.

**Figure 5:**
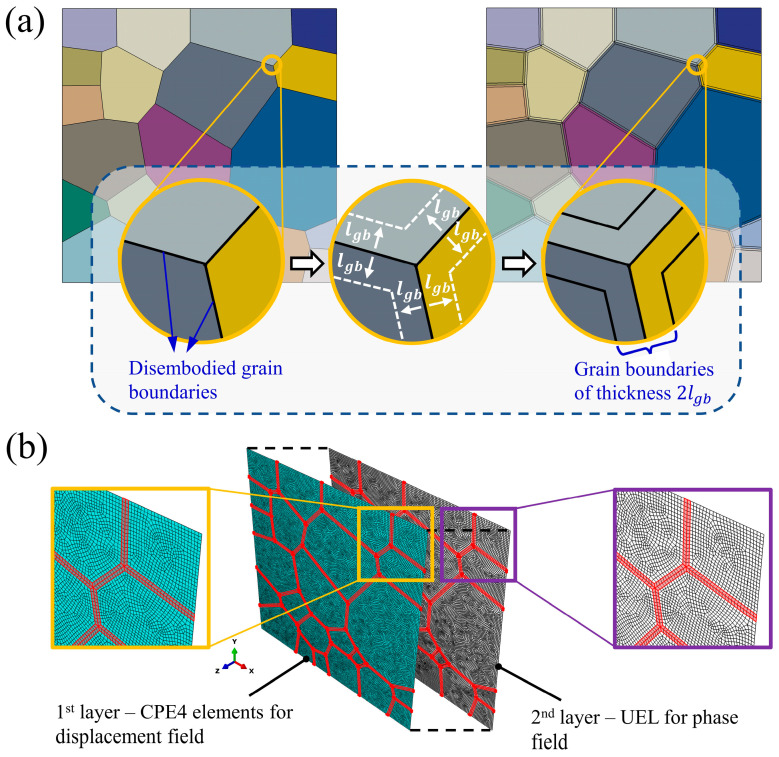
(**a**) Schematic of polycrystalline model construction and grain boundary generation process. (**b**) Illustration of the two-layer structure.

To capture the competing failure mechanisms between grain boundaries and grain interiors, a polycrystalline model with explicitly defined grain boundary structures was developed. The modeling process began with the generation of a grain structure using the NEPER software. The essential geometric information was subsequently extracted and reconstructed in the finite element software ABAQUS, enabling further modification of the crystal morphology. Boolean operations were applied to uniformly offset the boundaries of adjacent grains inward by a prescribed distance $l_{gb}$, thereby creating interstitial regions defined as explicit grain boundary volumes, as illustrated in Figure 5a. For computational clarity, all finite elements within the grain boundary domain were grouped into a set named GB-Set, as shown in Figure 5b. In this figure, the grain boundary elements are highlighted in red, while the remaining elements, representing the grain interiors, were assigned to the Inside-Set.

Since the fracture resistance of grain boundaries and grain interiors exhibits temperature dependence, it is essential to assign distinct failure thresholds to material points within these two regions. This is accomplished by specifying separate critical energy release rates, $G_{c}$, for the grain boundary and grain interior domains. These parameters define the energy required to initiate failure at a material point within their respective regions. The values of $G_{c}$ are informed by theoretical reference data obtained from subsequent molecular dynamics simulations.

### 3.3. Energy Determination for Failure

To theoretically elucidate the mechanisms of grain boundary decohesion and intragranular fracture, the critical energy release rates $G_{c}$ for material points at grain boundaries and within grain interiors are defined based on the grain boundary separation energy $W_{sep}$ and the surface energy $\gamma_{surf}$, respectively.

In the investigation of microscale fracture mechanisms in metallic materials, surface energy is a fundamental physical parameter that quantifies the energy required to generate a unit area of free surface by cleaving a perfect crystal along a specific crystallographic plane. For face-centered cubic (FCC) metals such as nickel (Ni), plastic deformation predominantly occurs via dislocation glide on the close-packed (111) planes along the [110] direction. Given the close association between fatigue damage and microscale instabilities on slip planes, the (111) plane is selected in this study as the representative crystallographic plane for evaluating intragranular fracture behavior. The corresponding surface energy is therefore adopted as the critical energy threshold for intragranular failure, i.e., $\gamma_{surf}=\gamma_{\left(111\right)}$, where $\gamma_{\left(111\right)}$ is the surface energy of the (111) crystallographic plane. This selection is also consistent with the constitutive assumptions of the crystal plasticity finite element model, in which plastic deformation is exclusively governed by shear slip in the [110] direction. The surface energy is computed using the following expression:

(19) $$\gamma_{\left(111\right)}=\frac{E_{surface}-E_{bulk}}{2A_{surface}},$$

where, $E_{surface}$ is the total energy of the system containing two free (111) surfaces; $E_{bulk}$ is the total energy of the unfractured system with the same number of atoms; and $A_{surface}$ is the surface area.

The grain boundary work of separation $W_{sep}$ is defined as the energy required to dissociate a perfect grain boundary into two free surfaces—i.e., to induce intergranular fracture—under ideal conditions without the involvement of external defects. It can be regarded as the intrinsic fracture strength of the grain boundary. According to the Rice–Wang model, the work of separation is calculated using the following equation:

(20) $$W_{sep}=\frac{2E_{FS}-E_{GB}}{2A_{GB}},$$

where $W_{sep}$ is the total energy of the system after grain boundary fracture, containing two newly formed free surfaces; $E_{GB}$ represents the total energy of the system with an intact grain boundary; $A_{GB}$ is the area of the grain boundary; and $E_{FS}$ denotes the total energy of the two newly generated free surfaces resulting from the fracture of a single grain boundary.

The grain boundary work of separation $W_{sep}$ and surface energy $\gamma_{\left(111\right)}$ were evaluated using molecular dynamics (MD) simulations conducted with the LAMMPS (https://www.lammps.org, accessed on 8 July 2025) software package. MD simulations provide an atomistic-level framework capable of accurately capturing microstructural evolution in material systems. In the context of grain boundary studies, MD allows the construction of idealized, impurity-free grain boundary models, thereby isolating the intrinsic cohesive behavior of the boundary from the effects of external contaminants. Furthermore, MD enables direct simulation of material responses under a wide range of conditions, including varying temperature, mechanical loading, and chemical environments. This makes it a particularly powerful tool for investigating grain boundary decohesion, calculating interfacial energies, and analyzing the fundamental mechanisms of microscale failure. As such, MD has been extensively employed in combination with crystal plasticity finite element methods (CPFEM) to examine mechanical behavior at the grain scale [64,65].

The calculation of the surface energy for the Ni (111) crystallographic plane follows a methodology similar to that used for evaluating the grain boundary work of separation, with some adjustments in model configuration and boundary conditions. The face-centered cubic (FCC) crystal structure of Ni was obtained from the ICSD database, and a slab model was generated using Atomsk (https://atomsk.univ-lille.fr/, accessed on 8 July 2025) by cleaving the crystal along the [111] plane. This model contains approximately 10,000 atoms, with a sufficiently large vacuum layer added along the thickness direction to eliminate interactions between the opposing fracture surfaces. Free boundary conditions were applied normal to the crystal plane, while periodic boundary conditions were maintained along the other two directions. Interatomic interactions were governed by the embedded atom method (EAM) potential for Ni developed by Mishin et al. [66]. The system was first pre-equilibrated under the NVT (constant volume and temperature) ensemble for 5 picoseconds, followed by relaxation under the NPT (constant pressure and temperature) ensemble for approximately 25 picoseconds to reach thermodynamic equilibrium. Once the total energy of the system stabilized, energy minimization was performed using the Steepest Descent method to obtain the slab model’s total energy (denoted as $E_{slab}$). A separate bulk crystal model with the same number of atoms and volume was constructed to calculate the bulk energy $E_{bulk}$. The surface energy of the Ni (111) plane at various temperatures was then derived using the previously defined expression. At 0 K (−273.15 °C), the calculated surface energy for the Ni (111) plane was found to be $1.95\;Jm^{-2}$, which closely aligns with reference values $1.92\;Jm^{-2}$ and $2.011\;Jm^{-2}$ reported in prior studies employing similar computational methodologies [67,68].

**Figure 6:**
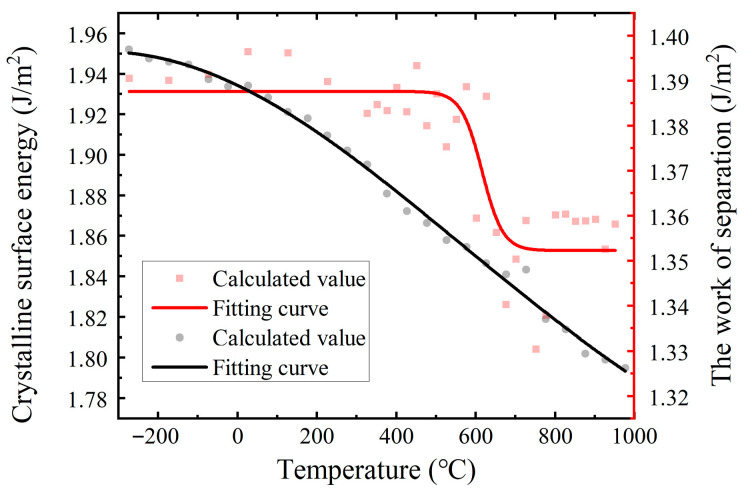
Theoretical values of grain boundary work of separation $W_{sep}$ and surface energy $\gamma_{\left(111\right)}$ at different temperatures.

To investigate the effect of temperature, molecular dynamics (MD) simulations were performed across a temperature range from room temperature to 1000 °C. Thermally equilibrated energy data were extracted for subsequent analysis. The values of grain boundary separation energy were adopted from the previous work conducted by our research group [69]. The results, presented in Figure 6, indicate that both surface energy and grain boundary separation energy exhibit a decreasing trend with increasing temperature in the range from room temperature to 750 °C. Notably, while the surface energy decreases approximately linearly with temperature, the grain boundary separation energy displays a two-stage behavior: it decreases gradually from room temperature to 600 °C, followed by a more rapid decline between 600 °C and 750 °C. This sharp drop in the latter interval suggests a pronounced weakening of the Σ5 grain boundaries in pure Ni, indicating a critical transition in interfacial cohesion within this temperature range.

**Figure 7:**
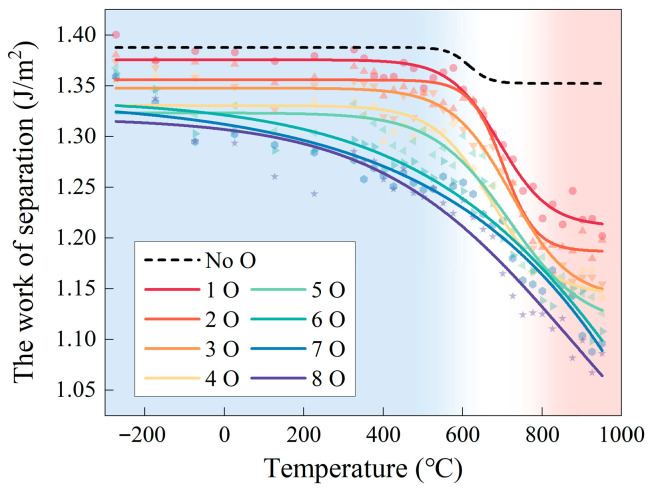
Grain boundary work of separation of Ni Σ5 grain boundary as a function of temperature. Reprinted from Ref. [69].

In addition to temperature, the presence of oxygen at grain boundaries is another critical factor that influences grain boundary strength. Oxygen atoms tend to accumulate at grain boundaries through interactions with the base metal, leading to a reduction in interfacial bonding strength. This process lowers the grain boundary cohesion energy and adversely affects the mechanical properties and fatigue life of the material [70]. The progressive diffusion and distribution of oxygen within the grain boundary region results in a continuous degradation of grain boundary strength, a phenomenon that can also be verified through molecular dynamics (MD) simulations. Zhao et al. developed a structurally optimized grain boundary model by introducing 1 to 8 oxygen atoms into the octahedral interstitial sites of a relaxed grain boundary structure prior to structural relaxation [69]. The temperature-dependent grain boundary separation energy of Ni Σ5 grain boundaries doped with varying oxygen concentrations is shown in Figure 7. The results demonstrate that the grain boundary separation energy decreases steadily with increasing oxygen content. Notably, grain boundary weakening becomes significantly more pronounced at temperatures above 600 °C.

### 3.4. Numerical Realization

To implement the coupling between the crystal plasticity constitutive model and the phase-field method in Abaqus/Standard, a dual-layer element framework was employed, as illustrated in Figure 5b. The first layer consists of standard CPE4 elements defined in Abaqus, each possessing two displacement degrees of freedom. Based on the nodal spatial coordinates of this layer, a second layer of user-defined elements (UELs) was generated within the model input file. These UELs share the same nodes as the first layer but are assigned a single degree of freedom corresponding to the phase-field variable.

Before entering the incremental loop, at the initial stage of the simulation, initial data are input, including the crystallographic orientations of the grains and material parameters required for both the crystal plasticity and phase-field models. These parameters are defined at the beginning of the computation and remain constant throughout the simulation process. Taking a single incremental step as an example, the computational procedure proceeds as follows: First, at each integration point of the first-layer elements, the phase-field value is retrieved from a common block and used to update the stiffness degradation function (Equation (15)). The UMAT subroutine is then called to compute the elastoplastic deformation and stress field, during which the stress tensor and stiffness matrix are degraded according to the weakening function. Subsequently, for each element in the second layer, the UEL subroutine is invoked. Key information, including the phase-field driving force, is again transferred via the common block, and the phase-field equation is solved within the UEL framework.

**Figure 8:**
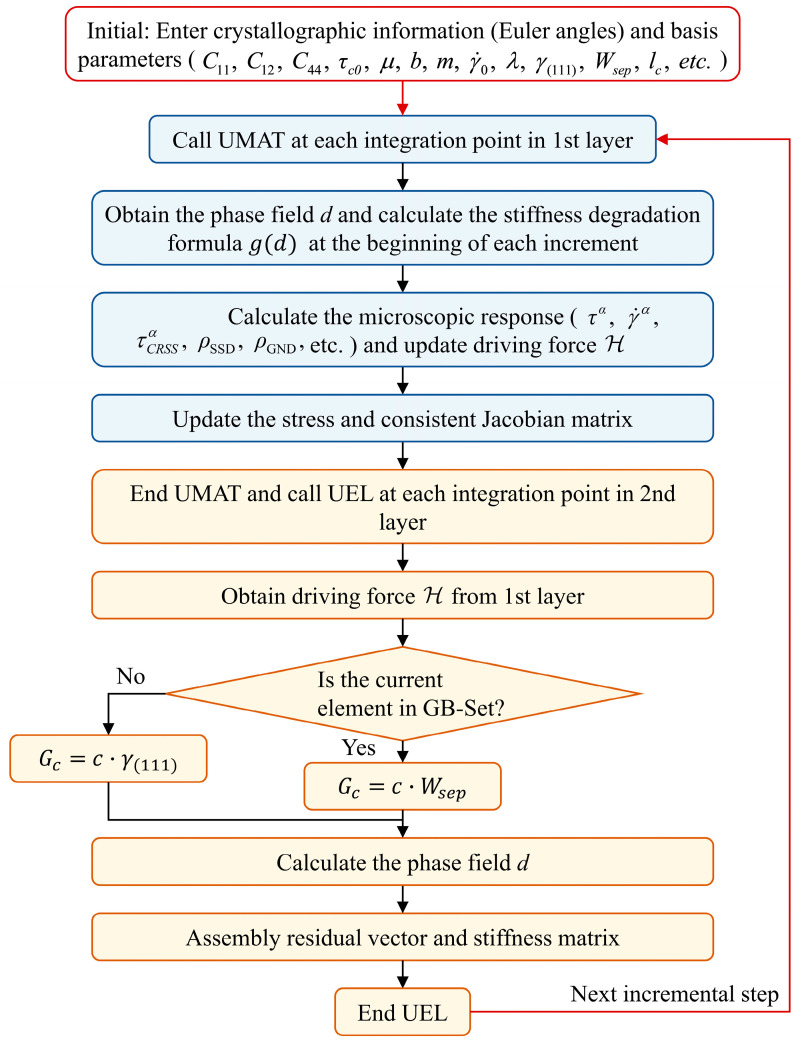
Computational workflow of the CP–PF model.

The model distinguishes between grain boundary and intragranular elements by indexing their element IDs. If the current element belongs to the GB-Set (i.e., the grain boundary region), the threshold value of the driving force required for failure, denoted as $G_{c}$, is assigned as a linear function of the grain boundary work of separation, $G_{c}=c\cdot W_{sep}$. Conversely, if the element belongs to the Inside-Set (i.e., the intragranular region), the corresponding threshold $G_{c}$ is assigned as a linear function of the surface energy, $G_{c}=c\cdot \gamma_{\left(111\right)}$. Finally, the current phase-field driving force is computed using Equation (14), which ultimately determines the evolution of the phase field. The overall computational procedure is illustrated in Figure 8.

## 4. Fatigue Crack Growth Simulation

### 4.1. Numerical Modelling and Results

**Figure 9:**
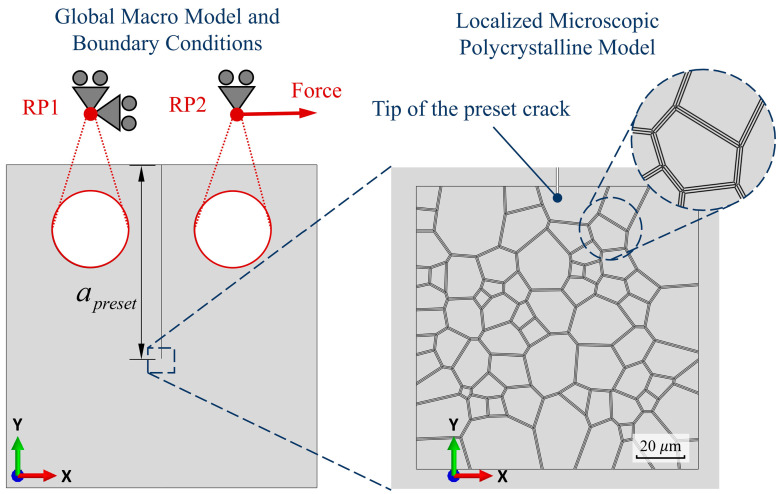
Schematic of the numerical model, including the macroscopic dimensions of the CT specimen, embedded microstructural region, and applied loading.

To investigate the fatigue crack growth behavior of the GH4169 alloy under different temperature conditions, a finite element model was constructed based on the geometry of a standard compact tension (CT) specimen. Three temperature scenarios were considered: room temperature (approximately 25 °C), a representative service temperature (650 °C), and an elevated temperature beyond typical service conditions (750 °C). The material parameters required for the simulations—including those for the crystal plasticity constitutive model and the critical energy release rates for intragranular and grain boundary failure (i.e., surface energy and grain boundary separation energy)—were assigned based on temperature-specific data. The overall modeling framework is illustrated in Figure 9, where a microscale polycrystalline structure is embedded at the tip of a pre-existing crack. The model is subjected to the same loading conditions as those applied in the corresponding fatigue crack growth experiments. During the simulation, integration points at which the phase-field variable exceeds 0.99 are considered fully damaged. At these points, the evolution of key state variables such as accumulated shear strain is terminated.

In addition, for each temperature condition, models incorporating varying degrees of grain boundary weakening were developed to investigate the influence of oxidation-induced grain boundary embrittlement on fatigue crack growth behavior. As both service temperature and exposure duration increase, grain boundary oxidation progressively intensifies. However, quantitatively assessing the extent of grain boundary weakening through experimental methods remains a significant challenge. As described earlier, this study employed molecular dynamics (MD) simulations to construct oxygen-doped grain boundary models and to calculate the corresponding theoretical values of grain boundary separation energy, which reflect different levels of interfacial degradation. Specifically, three representative values of grain boundary separation energy were adopted, corresponding to the NoO, 1O, and 8O cases in Figure 7, representing unoxidized, mildly oxidized, and moderately oxidized grain boundaries, respectively. Accordingly, a total of nine simulation models were established and designated as T25-NoO, T25-1O, T25-8O, T650-NoO, T650-1O, T650-8O, T750-NoO, T750-1O, and T750-8O, to facilitate subsequent analysis and discussion. To eliminate the potential disturbance caused by orientation differences between models, all nine models were assigned the same set of initial crystallographic orientations. These orientations were generated as fully random Euler angles using the open-source software Neper, representing a texture-free microstructure.

**Figure 10:**
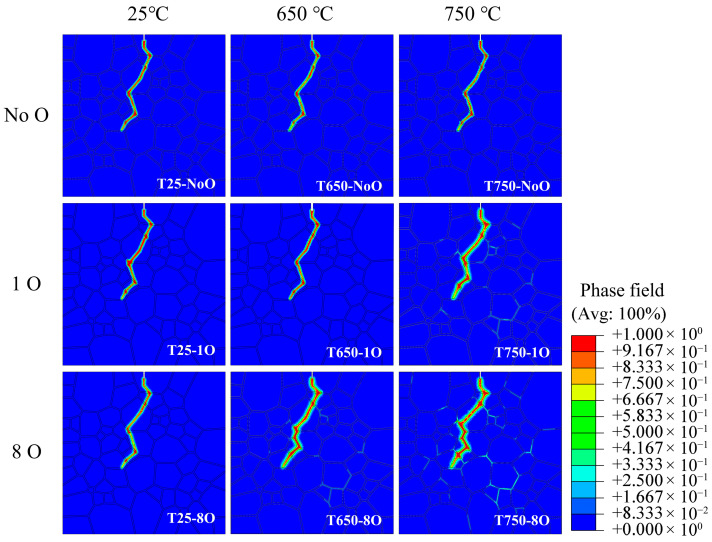
Simulated fatigue crack morphologies of all nine numerical models.

The crack morphologies of the nine simulation models were compared at a uniform crack length of 80 μm in the Y-direction. As shown in Figure 10, Under room temperature conditions, the crack paths exhibited notable similarity across different oxidation levels, all displaying predominantly transgranular propagation modes. Likewise, at elevated temperatures, the models without grain boundary oxidation (T650-NoO and T750-NoO) showed comparable crack growth patterns and retained transgranular characteristics. However, as the temperature increased to 650 °C and 750 °C, and the degree of grain boundary oxidation intensified, the crack paths began to exhibit more pronounced intergranular propagation features. Models such as T650-8O, T750-1O, and particularly T750-8O showed a noticeable increase in intergranular fracture behavior. The T750-8O model exhibited the highest intergranular crack propagation ratio (35.2%), calculated as the ratio of the intergranular crack length to the total length of the main crack path.

**Figure 11:**
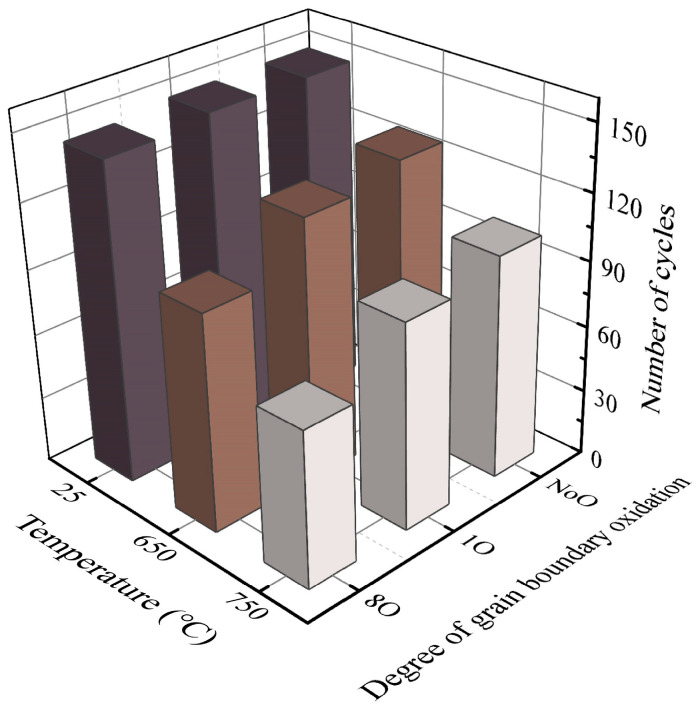
Number of cycles required for fatigue crack growth to a fixed length.

Figure 11 presents the number of loading cycles required for the crack to propagate to a fixed length in each simulation model. The results demonstrate that both elevated temperature and increased grain boundary oxidation significantly reduce fatigue life, although the extent of their effects varies across conditions. Specifically, for the room temperature cases, the number of cycles required for crack growth in models T25-NoO, T25-1O, and T25-8O were 149, 149, and 146, respectively, indicating that grain boundary oxidation had only a minor influence on fatigue crack growth resistance under ambient conditions. In contrast, as temperature increases, the fatigue life of oxidized models decreases more markedly compared to their non-oxidized counterparts. For instance, at 650 °C, the fatigue lives of models T650-1O and T650-8O were reduced to 94.4% and 77.9% of that of T650-NoO, respectively. Similarly, at 750 °C, the fatigue lives of T750-1O and T750-8O dropped to 92.2% and 69.6% of that of T750-NoO, respectively. These findings indicate that the detrimental effect of grain boundary oxidation on fatigue resistance is significantly amplified under high-temperature conditions.

### 4.2. Non-Oxidized Grain Boundary Analysis

We begin by analyzing the three models without grain boundary oxidation: T25-NoO, T650-NoO, and T750-NoO. As shown in Figure 10, the fatigue crack propagation paths in these oxidation-free models are nearly identical and exhibit predominantly transgranular fracture modes. It is noteworthy that this result does not fully align with observations reported in several experimental studies, which have identified a temperature-dependent transition in crack propagation modes—specifically, a tendency for fatigue cracks in high-temperature alloys to shift from transgranular to intergranular as temperature increases from room temperature to elevated levels [14,15,71,72]. In these simulations, the crack morphology displays a characteristic “Z-shaped” or zigzag propagation pattern, a feature commonly observed in other high-temperature alloys. For example, Yang et al. reported similar zigzag-shaped fatigue cracks in the FGH96 alloy at both room temperature and 600 °C, where the cracks tended to follow close-packed crystallographic planes [73]. This type of crack propagation has been shown to be primarily driven by the sustained accumulation of shear slip, which is highly sensitive to grain orientation [74,75,76].

**Figure 12:**
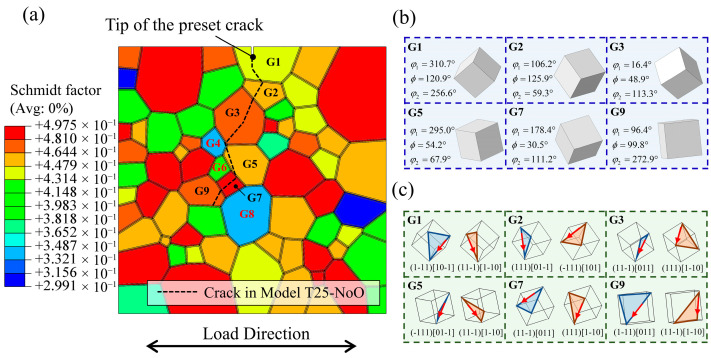
(**a**) Schmid factor distribution in the local polycrystalline model. (**b**) Grain orientation of cracked grains in oxygen-free model. (**c**) Activated close-packed planes and slip directions.

To elucidate the crack propagation mechanisms in the three oxidation-free models, the Schmid factors in the vicinity of the crack tip were extracted, as shown in Figure 12a. In this figure, the nearly identical fatigue crack paths from the three models are indicated by black dashed lines. The results show that the cracks consistently bypassed grains with relatively low Schmid factors—specifically, G4 (0.33), G6 (0.4), and G8 (0.34)—while preferentially propagating through grains with higher Schmid factors such as G2 (0.44), G3 (0.48), G5 (0.45), G7 (0.49), and G9 (0.46), ultimately forming the observed curved fatigue crack morphology. Furthermore, Figure 12b displays the crystallographic orientations of the cracked grains, while Figure 12c illustrates the most closely packed slip planes and the corresponding slip directions of the two most highly activated slip systems within these grains. The direction of crack propagation was found to be either aligned with or closely parallel to the slip direction of the primary activated slip system, further confirming the strong correlation between fatigue crack trajectories and grain orientation, as well as the slip-dominated nature of crack growth. These observations are consistent with previously reported fatigue crack propagation mechanisms at room temperature—and, in certain cases, at elevated temperatures—where sustained dislocation slip serves as the primary driving force for crack advancement.

The number of cycles required for the crack to reach the same unidirectional length in the three oxidation-free models—T25-NoO, T650-NoO, and T750-NoO—was 149, 127, and 102, respectively, demonstrating a significant decrease with increasing temperature. This trend reflects the general degradation of fatigue resistance at elevated temperatures.

**Figure 13:**
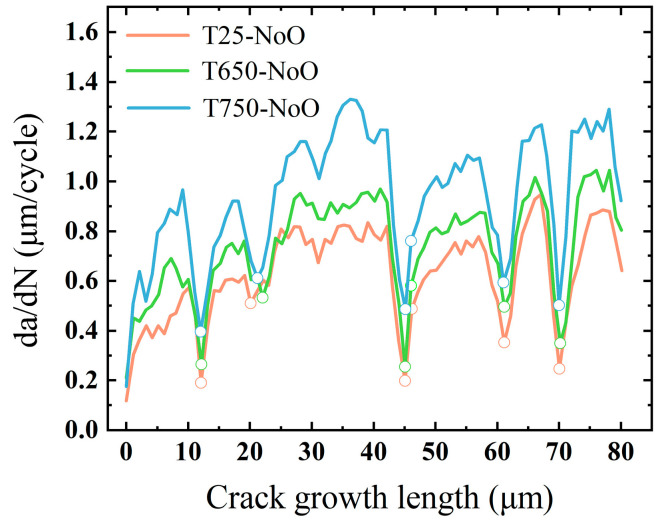
Fatigue crack growth rates in oxygen-free models.

Figure 13 presents the crack growth rate curves for the three models, where data points corresponding to grain boundaries are marked with hollow circles. Overall, the high-temperature models exhibit notably higher crack growth rates compared to the room-temperature model. A distinct reduction in crack growth rate is observed as the crack approaches grain boundaries, suggesting that—at all three temperature levels—grain boundaries, or more specifically, grains with misoriented crystallographic orientations, serve as barriers to fatigue crack propagation. This behavior is consistent with prior experimental findings, which reported similar reductions in crack growth rates near grain boundaries under cyclic loading conditions [77,78,79]. Studies by Tao et al. and MacLachlan et al. further indicate that this retardation effect is strongly influenced by the crystallographic characteristics of adjacent grains. In particular, when a crack approaches a grain with a low Schmid factor or encounters a high-angle grain boundary (i.e., a large orientation mismatch between neighboring grains), it experiences increased resistance and may even become temporarily arrested. In such scenarios, crack propagation may resume through coalescence with nearby secondary cracks or by transitioning to an alternate slip plane after several loading cycles [80,81].

### 4.3. Grain Boundary Oxidation Models Analysis

#### 4.3.1. Room Temperature Cases

To assess the influence of grain boundary oxidation on fatigue crack growth behavior at room temperature, three models—T25-NoO, T25-1O, and T25-8O—were analyzed. The crack paths in all three models were nearly identical, with transgranular propagation remaining the dominant fracture mode. As the degree of grain boundary oxidation increased, a slight reduction in the number of cycles required for the crack to reach the same length was observed, indicating a modest increase in crack growth rate.

**Figure 14:**
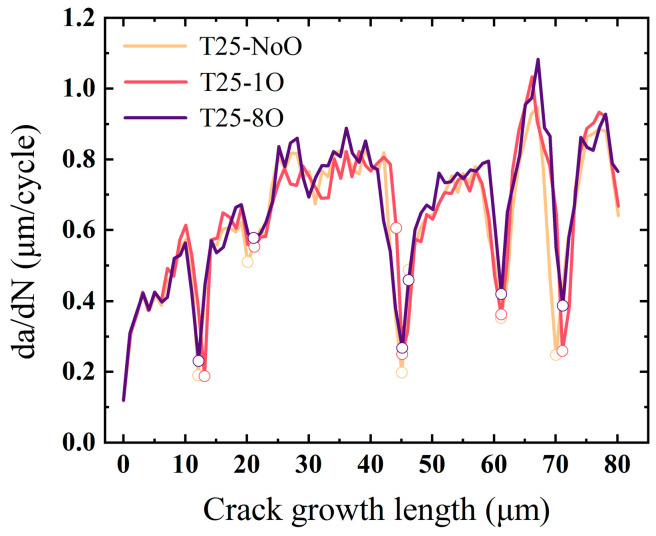
Fatigue crack growth rates of models with varying grain boundary oxidation levels at room temperature.

Figure 14 presents the fatigue crack growth rates for the three models. Across the majority of the propagation path—corresponding to intragranular regions—the crack growth rates are nearly identical. In all three cases, noticeable crack deceleration was observed as the crack tip approached grain boundaries, with the most pronounced variations in growth rate occurring in these regions. Notably, in the T25-8O model, the duration of crack tip arrest—measured by the number of cycles during which the crack remained near a grain boundary—was significantly shorter compared to the T25-1O and T25-NoO models. This indicates a reduction in crack propagation resistance at oxidized grain boundaries, even under room temperature conditions.

The above results can be attributed to the energy-based failure criteria defined within the model. As all simulations were conducted at room temperature, the three models shared identical mechanical parameters. Given that oxidation has a negligible effect on intragranular regions at this temperature, the surface energy associated with intragranular fracture remained constant across all models. Therefore, the only variation among the three lies in the grain boundary work of separation. When a crack approaches a grain boundary, the misorientation between adjacent grains governs the boundary’s resistance to crack propagation. However, with increasing levels of grain boundary oxidation, the separation energy in models T25-1O and T25-8O decreased by 1.19% and 5.93%, respectively, relative to the T25-NoO model. As a result, under nearly identical driving forces, grain boundaries in the oxidized models experienced more pronounced damage and reduced cohesion, thereby diminishing their ability to hinder crack propagation and shortening the crack tip dwell time. In contrast, since the surface energy parameter for intragranular elements was held constant, the resistance to crack propagation within individual grains remained effectively unchanged across the three models. This explains the observed consistency in crack growth rates during intragranular propagation. In summary, for room-temperature conditions, differences in fatigue life are primarily governed by the degradation of grain boundary strength due to oxidation. This conclusion is consistent with the experimental findings of this study, where longer thermal exposure—corresponding to more severe oxidation—led to accelerated fatigue crack growth rates.

#### 4.3.2. Elevated Temperature Cases

**Figure 15:**
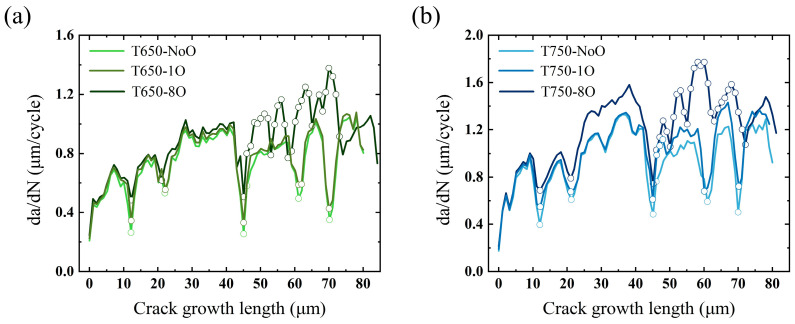
(**a**) Fatigue crack growth rates of models at 650 °C. (**b**) Fatigue crack growth rates of models at 750 °C.

This section analyzes the models incorporating oxidized grain boundaries under high-temperature conditions. As shown in the simulation results in Section 4.1, the proportion of intergranular crack propagation in the T750-1O and T750-8O models increased significantly compared to the low-temperature cases, indicating a temperature-driven transition in crack propagation mode from transgranular to intergranular. Figure 15 presents the fatigue crack growth rate curves for the four high-temperature oxidation models, alongside data from their non-oxidized counterparts at the same temperatures for comparison. Similar to the observations at room temperature, the fatigue crack growth rates within grains in the T650-1O and T750-1O models show no substantial increase relative to the T650-NoO and T750-NoO models, respectively. In the T750-1O model, a localized intergranular segment approximately 5 μm in length was observed. Although the crack growth rate within this region was marginally higher than in the corresponding region of the T750-NoO model, its limited spatial extent resulted in a negligible impact on the overall fatigue life. Therefore, the observed reduction in fatigue life can be primarily attributed to the decreased resistance to crack propagation near grain boundaries, arising from oxidation-induced reductions in grain boundary work of separation.

It is worth noting that the T650-8O and T750-8O models exhibited the most pronounced acceleration in crack growth. In Figure 15, all segments of the crack paths that coincide with grain boundaries are marked with hollow circles. Both models clearly display dominant intergranular propagation modes, and within these segments, the crack growth rate is significantly higher than that observed during transgranular propagation. Therefore, in addition to the diminished crack-arresting capability of grain boundaries due to oxidation, the inherently faster propagation kinetics associated with intergranular cracking represents another key factor contributing to the substantial reduction in fatigue life observed in these models. This further reinforces the strong correlation between grain boundary oxidation and accelerated fatigue crack growth behavior.

**Figure 16:**
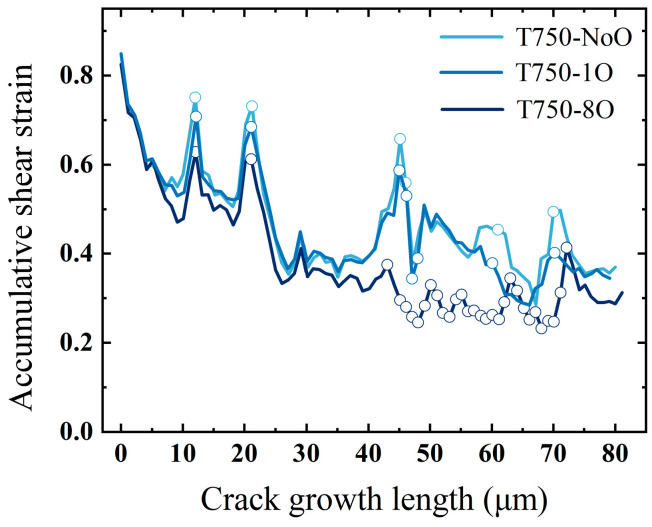
Total shear strain along fatigue crack paths for models with different grain boundary oxidation levels at 750 °C.

To gain deeper insight into the underlying mechanism, the cumulative shear strain along the crack paths in the T750-1O and T750-8O models at 750 °C was extracted and is presented in Figure 16. The results reveal that total slip system activation in the intergranular segments—highlighted by circles—is markedly lower than in the transgranular regions. This suggests that, under the combined effects of elevated temperature and severe grain boundary oxidation, the structural weakening of grain boundaries substantially reduces the energy barrier for intergranular crack propagation. Consequently, the crack growth mode progressively transitions away from transgranular propagation—primarily driven by dislocation slip—toward intergranular propagation, which emerges as a more energetically favorable pathway under these conditions.

**Figure 17:**
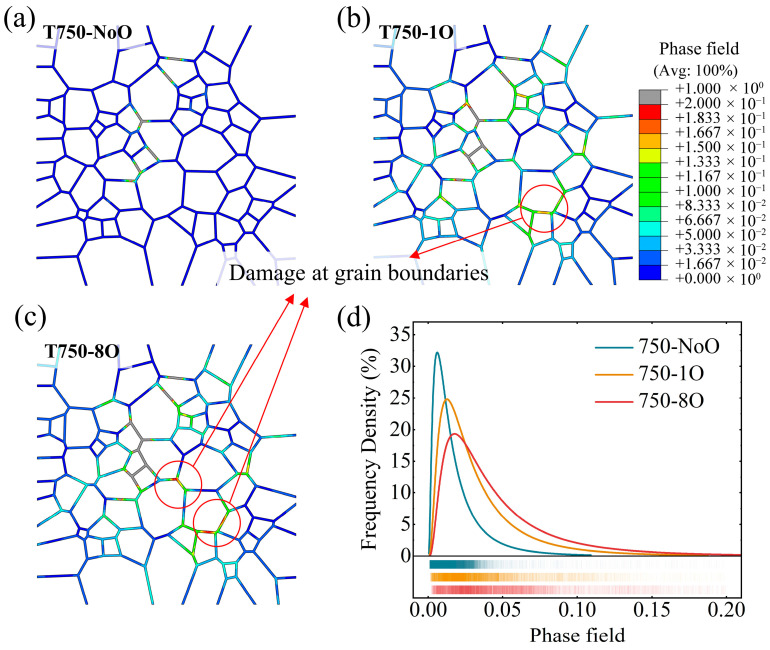
Grain boundary phase field and frequency distribution for (**a**) model T750-NoO, (**b**) model T750-1O, and (**c**) model T750-8O at 750 °C. (**d**) The frequency distribution of grain boundary phase field values except for the crack area.

In addition to the main crack path, signs of secondary intergranular crack initiation were observed in the T750-8O model. Figure 17a–c show the grain boundary phase-field distributions for the T750-NoO, T750-1O, and T750-8O models at 750 °C, when the fatigue cracks had propagated to the same unidirectional length, thereby highlighting the extent of grain boundary degradation. The average phase-field values at grain boundary integration points in the three models were calculated as 0.029 for T750-NoO, 0.072 for T750-1O, and 0.097 for T750-8O, further confirming the oxidation-induced weakening of grain boundaries, which is particularly severe in the T750-8O model. By excluding the severely damaged segments corresponding to the primary crack path (the area with d<0.2 and indicated in gray), it becomes evident that the remaining grain boundaries in the T750-1O and T750-8O models exhibit higher overall damage levels compared to the non-oxidized model. The frequency distributions of grain boundary phase-field values are summarized in Figure 17d. As the degree of grain boundary oxidation increases, damage accumulation along the boundaries becomes progressively more severe, with widespread degradation and an increasing tendency toward grain boundary failure. These simulation results are consistent with experimental observations at elevated temperatures, which have shown that, under high-temperature fatigue conditions, grain boundaries oriented parallel to the main crack path frequently undergo oxidation-induced decohesion and serve as preferential sites for secondary crack initiation [82,83]. Therefore, in high-temperature service environments where superalloys are exposed to oxidizing atmospheres, the risk of grain boundary decohesion and multipoint damage—manifesting as secondary or branching cracks—is significantly heightened. These phenomena pose serious challenges for maintaining the structural integrity and fatigue reliability of the material.

### 4.4. Model Limitations and Future Improvements

Despite the valuable insights offered by the numerical model developed in this study into the micro-mechanisms of high-temperature fatigue crack growth, several important limitations remain. First, the grain boundary work of separation used to simulate the effects of oxidation was derived from a predefined set of theoretical values. While these parameters provide a reasonable basis for comparative analysis, they do not fully capture the complex, spatially heterogeneous damage states induced by grain boundary oxidation in real materials. Establishing a unified and quantifiable relationship between the degree of oxidation and the actual mechanical degradation of grain boundaries remains a key direction for future investigation. Second, the current model does not incorporate dynamic oxidation processes occurring during crack propagation. Instead, each sub-model assumes a fixed oxygen content at the grain boundaries, corresponding to pre-assigned degradation levels. This assumption aligns with the experimental protocol used in this study’s room-temperature fatigue tests, wherein fatigue resistance was evaluated after thermal aging, thus excluding real-time oxidation at the crack tip. However, such simplification limits the model’s applicability to realistic service environments involving simultaneous mechanical loading and environmental exposure at elevated temperatures. Future research could address this limitation by introducing oxidation reaction kinetics into the simulation framework. One possible strategy would be to embed a damage evolution model dependent on temperature and oxygen concentration within the crystal plasticity–phase field fracture (CP–PFF) framework. Alternatively, drawing on the chemo-mechanical coupling approach proposed by Li et al. for hydrogen-assisted cracking [63], the present model could be extended to include a phase-field module for oxygen diffusion. These enhancements would significantly improve the predictive capability of the model under more realistic service conditions.

## 5. Conclusions

To investigate the impact of grain boundary weakening on the fatigue resistance of nickel-based superalloys, this study combined both experimental and numerical approaches. Room-temperature fatigue crack growth experiments were performed on GH4169 alloy specimens subjected to various aging temperatures and durations. The results demonstrated that with increasing aging temperature and prolonged exposure time, grain boundary oxidation became progressively more pronounced, resulting in a reduction in fatigue life and an earlier transition in crack propagation mode from transgranular to intergranular.

On the numerical side, a crystal plasticity–phase field fracture (CP–PFF) model incorporating explicitly defined grain boundary structures was developed. The grain boundary work of separation and surface energy—obtained via molecular dynamics simulations—were employed as critical energy thresholds for the failure of grain boundary and intragranular material points, respectively. This framework enabled the model to capture the competition between transgranular and intergranular crack growth mechanisms with high fidelity. A total of nine simulation scenarios were constructed, covering three temperature conditions (room temperature, 650 °C, and 750 °C) and three oxidation levels, thereby facilitating a systematic investigation of fatigue crack growth behavior. The main conclusions of this study are summarized as follows:

- Both elevated temperature and increased grain boundary oxidation contribute to a reduction in fatigue life. The simulation results successfully reproduced the experimentally observed variations in fatigue life and crack propagation morphology under different thermal and aging conditions, demonstrating the model’s sensitivity to changes in temperature and grain boundary oxidation levels.
- In the absence of grain boundary oxidation, fatigue crack growth at both room and elevated temperatures is governed by continuous dislocation slip. Cracks predominantly follow the direction of the most highly activated slip system. Variations in grain orientation, particularly the presence of grains with low Schmid factors, act as barriers to crack propagation.
- At room temperature, oxidation-induced reduction in grain boundary work of separation leads to shorter crack arrest durations at grain boundaries and a moderate decrease in fatigue life. However, the dominant crack propagation mode remains transgranular.
- At elevated temperatures, oxidation significantly degrades grain boundary toughness, thereby reducing the resistance to crack propagation and promoting intergranular fracture. This results in a more pronounced decrease in fatigue life. Furthermore, the accumulation of damage near the crack tip is intensified, with observable signs of secondary intergranular crack initiation.

## Figures and Tables

**Table 1:** Crystal plasticity material parameters of GH4169 alloy at RT, 650 °C, and 750 °C.

Temperature	25 °C	650 °C	750 °C
$C_{11}(GPa)$	267	191	153
$C_{12}(GPa)$	114.5	88	66
$C_{44}(GPa)$	76	66	50
μ(GPa)	75	67	50
${\dot{\gamma }}_{0}\;(s^{-1})$	0.002	0.0014	0.001
$\tau_{c0}(MPa)$	390	317	290
b (μm)	$2.54\times 10^{-4}$	$2.54\times 10^{-4}$	$2.54\times 10^{-4}$
$\lambda \;({\mu m}^{-2})$	82	74	56
m	0.02	0.051	0.068

**Figure A1:**
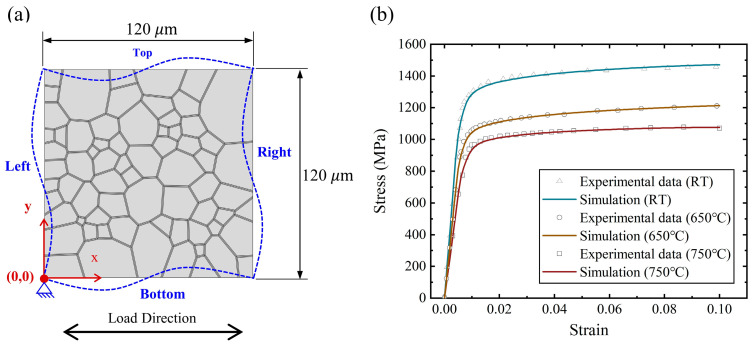
(**a**) Schematic illustration of the representative polycrystalline model and the boundary conditions for uniaxial tensile loading. (**b**) Comparison of stress–strain curves obtained from uniaxial tensile experiments and calibrated CPFE simulations at 25 °C, 650 °C, and 750 °C.

## Data Availability

The original contributions presented in the study are included in the article, further inquiries can be directed to the corresponding author.

## Appendix A

To accurately capture the elastoplastic behavior of GH4169 superalloy under different temperature conditions, the material parameters required for the crystal plasticity finite element (CPFE) simulations were calibrated through a combination of uniaxial tensile experiments and numerical simulations. The experimental setup, including the tensile testing machine and specimen dimensions, was consistent with our previous study [84]. The tensile tests were conducted at 25 °C, 650 °C, and 750 °C, yielding true stress–strain curves for each condition, which served as the reference data for parameter fitting.

A representative polycrystalline model with an average grain size of 15 μm was constructed, containing explicit grain boundaries. The crystallographic orientations of the grains were assigned randomly to eliminate the influence of texture on the calibration results. Periodic boundary conditions were applied to simulate uniaxial tension, as illustrated in Figure A1a. Following the CPFE framework described in Section 3.1.1, numerical simulations were carried out, and the simulated stress–strain curves were iteratively compared with experimental data.

Through this iterative procedure, a set of elastoplastic parameters was determined for each temperature condition to best match the experimental responses. These calibrated parameters are summarized in Table 1 of the main text. Figure A1b presents the comparison between the CPFE simulation results and the experimental curves, demonstrating good agreement.

Further details regarding the numerical implementation and optimization strategy used in the calibration process can be found in our previous publication [84].
